# Revolutionizing healthcare leadership the critical role of digital citizenship in knowledge sharing

**DOI:** 10.1038/s41598-025-93117-y

**Published:** 2025-03-15

**Authors:** Naveed Saif, Sadaqat Ali, Imrab Shaheen, Gerald Guan Gan Goh, Sajid Ullah Khan

**Affiliations:** 1 Institute of Management Science, University of Science and Technology Bannu, Bannu, Pakistan; 2 National University of Modern Languages, Rawalpindi, Pakistan; 3 University of Kotli, Azad Jammu and Kashmir, Kotli, Pakistan; 4 Faculty of Business, Multimedia University, Melaka, Malaysia; 5 Information Systems Department, College of Computer Engineering and Sciences, Prince Sattam bin Abdulaziz University, Alkharj, Saudi Arabia

**Keywords:** Digital leadership, Visionary leadership, Knowledge sharing, Digital citizenship behavior, Healthcare workers, Psychology, Human behaviour

## Abstract

This study explores the relationship between digital leadership (DLR), visionary leadership (VSL), and knowledge sharing (KNS) among healthcare professionals in Pakistan, focusing on the mediating role of digital citizenship behavior (DCZ). As leadership becomes increasingly crucial for promoting knowledge sharing in healthcare, understanding how DLR and VSL facilitate this behavior particularly through digital citizenship is essential. A quantitative approach was employed, gathering data from 202 healthcare professionals via a structured questionnaire assessing DLR, VSL, DCZ, and KNS. Structural equation modeling (SEM) was used to analyze the relationships and test the mediating effect of DCZ. Findings reveal significant positive relationships between both DLR and VSL with KNS. Notably, DCZ partially mediates the relationship between DLR and KNS, with a stronger mediation effect observed in this context. In the VSL-KNS relationship, DCZ’s mediation was confirmed and indicating partial mediation. These results underscore the importance of fostering digital citizenship behaviors to enhance knowledge sharing, providing valuable insights for leadership development programs aimed at improving organizational performance in healthcare settings.

## Introduction

The role of technology cannot be overemphasized as it helps facilitate knowledge management in organizations^1^ especially with the factor being supported by strong visionary leadership that encourages technology adoption^2^. In today’s competitive environment, communication and information technology help people to share information with one another and geographical barriers are no longer an issue^1^. Applications like cloud technologies, employee social networks, and collaborative software and tools, help the employees of different organizations, departments and geographical locations to participate and share their knowledge^3^ and ideas with all the others instantly, thus helping in fostering transparency and inclusiveness. Hence, there is the need for adopting digital leadership^4^ that enables one to use these technologies appropriately in order to foster this knowledge-sharing ecosystem^5^. Successful leaders who understand the culture of digital era design organizations in which technology is not an instrument but the key means to facilitate learning and discovery. Digital leadership in healthcare can be express as “*Digital leadership in healthcare refers to the strategic use of digital technologies and data-driven innovations by leaders to enhance patient care*,* streamline operations*,* and drive organizational transformation*.”

In addition, visionary leadership also facilitates the implementation of knowledge management systems that creates ways of acquiring, organizing, and disseminating organizational knowledge^6^. Managers with a digital mindset know that knowledge sharing is implemented based on corporate values and practices leading to supporting the organization goal^7^. In this regard, there is encouragement of free flow of ideas where employees can contribute to what is going on, with the expectations being made clear that their inputs will be welcomed and taken into consideration^8^. Visionary leadership can be expressed as *“Visionary leadership in healthcare involves inspiring and guiding teams toward a shared*,* future-focused goal of improving patient outcomes*,* fostering innovation*,* and driving systemic change while adapting to emerging challenges and opportunities in the healthcare landscape.”*

Furthermore, the technology based leadership styles call for the provision of feedback in real time so that the leaders are able to recognize any voids that may be ailing the sharing of knowledge^5^^,^^4^. Through social media, face-to-face meeting apps, and other communication tools, it is also possible to create more involved and connected employees^9^. When these tools are used, executives endeavor to promote flexibility, sharing of ideas, knowledge, and enterprise resources through technological interventions resulting from typical visions for competitiveness and better outcomes^10^. Similarly, digital leadership promote the application of new technologies i.e. meta verse^11^, big data, machine learning^12^ and algorithmic approach to enhance employees learning and promote the culture of knowledge sharing^13^. Hence, it is critical to examine that how the interaction between technology and visionary leadership^6^ in an organization can be key contributor for the optimization of knowledge sharing in a knowledge-intensive context of the contemporary world to promote the concept of Digital Citizenship^14^.

Digital Citizenship (DC) covers behaviors and attributes, which are aimed at creating and promoting appropriate technology settings and connections^15^. It relates to peoples’ interactions with technologies in manners that stimulate new forms and processes that enhance innovation within the standards of ethics, laws and societal norms. In the context of educational institutions, leaders have a central role to play in defining and sustaining this kind of digital culture^16^. They are also engaged in the process of teaching learners and the staff how to use technologies safely and appropriately, and what users’ rights and responsibilities are when interacting with technologies.

The institutional leaders are required to promote the consciousness of ethical issues in the cyberspace including privacy, data security, and cyber threats. They also need to promote adherence to legal standards that regulate social use and interaction in the cyberspace and make users understand legal consequences of their conducts in the cyberspace^17^. In addition, the leaders are required to foster the development of appreciation of the societal obligations inherent in the cyber community. This entails practicing & encouraging civil, diversity healthy referring, manners, & equity in the realm of social media, thus creating harmony for every individual in the online society^18^.

Furthermore, there are other major ethical and social responsibilities of leaders to ensure that learners and staff are equipped adequately for the modern digital-world from a technological perspective^17,19^. This entails ensuring that there is adequate facilities that supports it especially in the area of infrastructure for the needed tools like internet, gadgets and user-friendly systems, AR/VR^20^facilities for learning and sharing information. Leaders must also ensure they invest into advance training to enhance their HR capabilities through application of modern technologies.

Ultimately, leadership plays a pivotal role in cultivating a responsible digital culture, where technology is leveraged for growth and learning while ensuring ethical, legal, and social obligations are met^4,5,14^.

Digital and visionary leadership play crucial roles in facilitating knowledge sharing and digital transformation in healthcare. Leaders need to develop new skills and attributes to effectively navigate the digital landscape, including adaptability, innovation, and a forward-looking perspective^4–6,14^.

In an increasingly digitized healthcare landscape, leaders who can foresee the transformative potential of technology and implement it effectively are essential for improving both patient care and organizational efficiency. Visionary leaders, with their forward-thinking mindset, foster an environment where innovation and technology integration are not only encouraged but also strategically aligned with the long-term goals of the healthcare organization^8,21^.

The digital transformation of healthcare encompasses various technologies, such as e-Health, m-Health, and telemedicine, which require leaders to employ strategies like systems thinking and contextual intelligence^22^. Leadership styles significantly impact knowledge-sharing behavior among followers, with different styles affecting individual and team-level outcomes^1,5,23,24^. The COVID-19 pandemic has accelerated the adoption of digital technologies in healthcare, leading to changes in management and business practices^20^.

Visionary leadership plays a crucial role in shaping the digital culture by emphasizing the importance of knowledge sharing^2^. These leaders understand that by breaking down silos and encouraging open communication across departments, healthcare organizations can improve patient outcomes and reduce inefficiencies. On the other side Digital leaders^4,5,14^ also make sure that the requite technologies such as communication technologies, cloud technologies, social media, collaboration tools and others are provided to support the flow of knowledge. Digital leadership promotes the concept of digital citizenship in that apart from having skills and knowledge on use of technological tools in their area of practice^14^, they also understand and appreciate the ethical concerns in the use of technology for instance in sharing patient information.

When visionary and digital leadership practices are implemented jointly, there will be improvement of learning climate and collaboration within healthcare organizations to allow for the production of better patient outcomes and overall organizational performance. It is hereby evident that visionary^6,8,21^ and digital leadership^4,5,14^ is instrumental in the sharing of knowledge^14^ in health care through developing of digital citizenship^16^. Visionary leaders are the key who introduce forward-thinking ways how to leverage digital assets to increase patients’ benefits and make processes more effective and optimized. For instance the study of^25^ proposed novel approach of CardioGaurd that offers superior performance in both security and patient record. By adopting technologies such as AI, telemedicine, and VR/XR/AR^20^, they remove the barriers in knowledge sharing thereby facilitating real-time exchange of data and information across the healthcare departments^7^. This is something for which digital leaders can support by guaranteeing the structures as well as the understanding required for efficient use of these tools. Combined, they promote digital citizenship^14^ where healthcare professionals are equipped with best practices on sharing of knowledge to improve on patients’ outcome as well as the overall organizational performance. The results from the study of indicate that visionary^8^ and digital leadership^26^ enhance employees performance in and also promote the culture of knowledge sharing^5^. Such an environment foster the feeling of digital citizenship behavior^15^ among workers, that nature the seeds for organizational overall effectiveness through innovation, credibility and improved performance as well as organizational goodwill.

From a theoretical prospective, digital leadership refers to innovative use of technology, enables employees to be empowered through technology to embrace transparent communication and collaboration in order to enhance knowledge sharing^27^. Visionary leadership that effectively provides employees with inspiring vision and future direction that helps to link their personal interests with organizational goals in order to increase intrinsic motivation for proactive knowledge sharing^28,29^support it. Organizations employee’s citizenship behavior, which refers to behaviors performed by employees in addition to their formal roles, appears where leadership fosters trust and delegation. This behavior is even more profound when the employees understand that they are part of a team with a common vision like the one provided by visionary leaders, and quality digital support from leaders through digitization. Both together maintain the organizational culture in which people appreciate, and use knowledge proactively to share it without pressures, and at the same time improve the innovation and learning processes resulting from successful cooperation^30^.

Previous research has extensively explored how digital and visionary leadership individually contribute to knowledge sharing (KNS). Digital leadership focuses on the practical aspects of technology and its implementation, while visionary leadership emphasizes long-term goals and innovation. Additionally, the literature has examined how digital citizenship responsible and ethical use of digital tools affects knowledge sharing. However, there is a noticeable gap in the literature regarding the application of these concepts specifically within the healthcare sector. In the context of Pakistan’s health care system, it is now even more important for digital and visionary leadership development to address difficulties like the lack of resources, unproductiveness, and population needs for proper treatment. Digital leadership helps in managing change by embracing technology, while visionary leadership transforms employees by sharing a destiny. Combined, these leadership styles may effectively encourage the knowledge-sharing behaviors of employees, which are crucial for organization learning and enhanced health care. Nevertheless, researchers still pay insufficient attention to the processes underlying digital and visionary leadership on knowledge-sharing behavior, especially, operating through the lens of digital citizenship behavior. Digital citizenship behavior, which is the proper and constructive interaction with different tools and platforms, is essential for efficient knowledge sharing in the healthcare setting. However, the findings reveal a scarcity of research on how these leadership styles work collectively to foster such behaviors, particularly in a country developing like Pakistan. This gap prevents healthcare organizations from being able to develop drives and plans that can utilize leadership in promoting the sharing of knowledge. Thus, this research intends to explore the influence of digital and visionary leadership on digital citizenship behavior, knowledge sharing behavior among the employees of selected healthcare organizations in Pakistan to offer operational recommendations for improving the service delivery and organizational effectiveness of the sector.

**Fig. 1:**
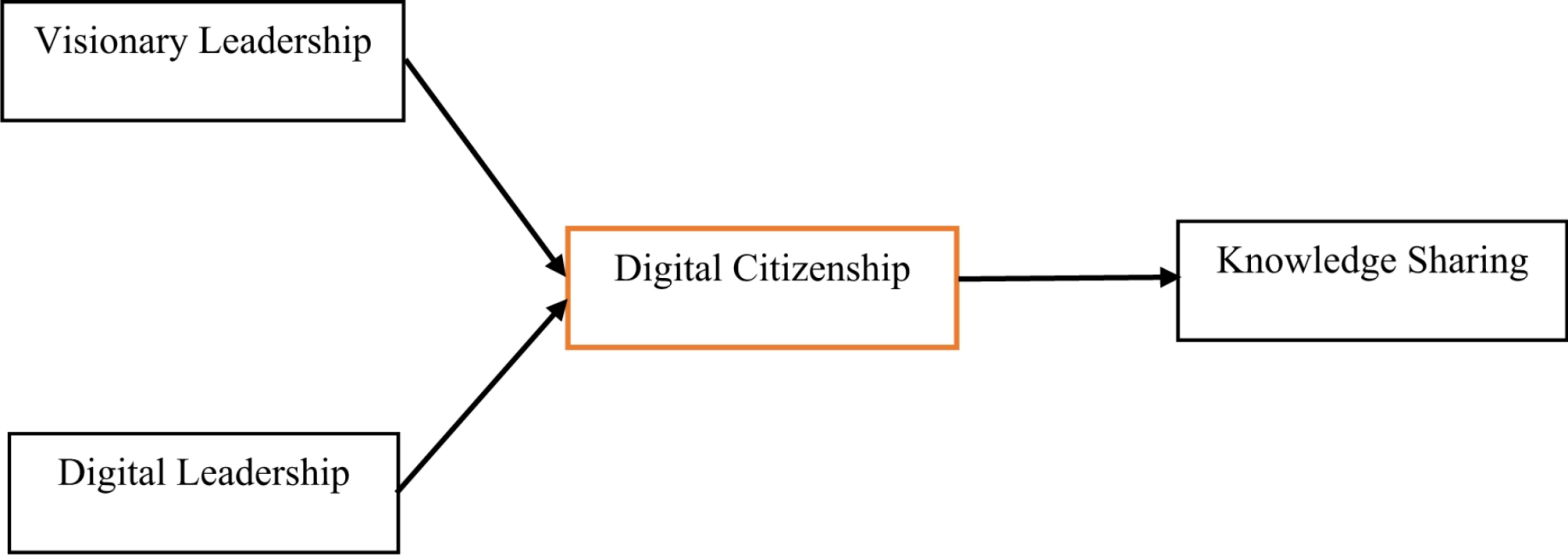
Conceptual Framework. Conceptual Model.

The current study addresses this gap by proposing a novel framework that integrates both digital and visionary leadership to enhance knowledge sharing through the promotion of digital citizenship (see Fig. 1).This approach is innovative because it combines two critical leadership styles in a way that has not been previously explored in the context of healthcare. By aligning these leadership attributes, the study provides a new perspective on how healthcare leaders can foster a culture of digitalization and knowledge sharing.

This research offers valuable insights into how healthcare organizations can effectively use both digital and visionary leadership to improve their knowledge sharing practices. In the era of the Fourth Industrial Revolution, where digital transformation is accelerating, this combination of leadership styles can help healthcare institutions adapt and thrive. By promoting responsible digital practices and creating a vision for technological advancement, healthcare leaders can better support their teams and enhance organizational performance. This study highlights the importance of integrating these leadership approaches to prepare healthcare institutions for future challenges and ensure they remain competitive in a rapidly evolving industry.

Based on the above discussion below mentioned are the research hypothesis to be investigated in the current study;

- H1; Digital citizenship promote knowledge sharing among health workers in Pakistani healthcare workers’ context.
- H2; Digital leadership in health sector has significant relationship with promoting digital citizenship among workers in Pakistani healthcare workers’ context.
- H3; Digital leadership in health sector has significant relationship with promoting knowledge sharing among workers in Pakistani healthcare workers’ context.
- H4; Visionary leadership in health sector has significant relationship with promoting digital citizenship among workers in Pakistani healthcare workers’ context.
- H5; Visionary leadership in health sector has significant relationship with promoting knowledge sharing among workers in Pakistani healthcare workers’ context.
- H6; Digital leadership in health sector mediate the relationship between digital citizenship and knowledge sharing among workers in Pakistani healthcare workers’ context.
- H7; Visionary leadership in health sector mediate the relationship between digital citizenship and knowledge sharing among workers in Pakistani healthcare workers’ context.

## Materials and methods

The population of this study consists of employees working in the healthcare sector. These individuals are expected to provide critical insights into the role of visionary and digital leadership in promoting knowledge sharing through digital citizenship within healthcare organizations. The sample size for this study was determined based on the guidelines provided by Hayes et al. (2023), which recommend obtaining 10 responses for each survey item. Given that the current study involves four variables with 18 items, the minimum required sample size for data analysis is 180 respondents (18 items * 10 response per item = 180 responses). This approach ensures that the sample size is adequate for conducting robust data analysis using Smart PLS (Partial Least Squares Structural Equation Modeling). Questionnaires were distributed among 350 respondents and after one month, the response rates indicate 220 valid construct with 18 responses with missing values and being incomplete. After removing the questionnaire that had missing values, a final response set of 202 were retained for further analysis. It is worth mentioning to resolve the issue of common method variance (CMV) data was collected in dyads. In this procedure employees rate about their leaders’ attributes related to digital and visionary capabilities while performing their job. On the other side after interval of 20 days’ relevant leaders responded about employee digital citizenship and their knowledge sharing attributes during performing their job.

The data for this study was collected using a structured questionnaire, comprising adapted constructs from established scales. The key variables under investigation are as follows:

Knowledge sharing (KNS): This variable was measured using the items adapted from the instrument developed by Donate and De Pablo^31^. This scale assesses how healthcare employees share and disseminate knowledge within their organizations.

Visionary leadership (VSL): The Principal Technology Leadership Assessment, developed by ISTE and adapted by Al Ajmi^32^ as well as recently validated in education set up^33^ and used by^5^ was used to measure digital leadership.

Digital citizenship (DCZ): DCZ was measured using four items adapted from Anwar and Saraih^5^. These items focus on how institutional heads promote access to appropriate digital tools and resources, as well as the ethical and responsible use of technology.

Digital leadership in healthcare (DLR): This variable was assessed using the scale developed by Claassen et al.^34^, which measures how digital leadership is operationalized within the healthcare sector. All responses were collected using a 5-point Likert scale, ranging from 1 (strongly disagree) to 5 (strongly agree).

The collected data was analyzed using Structural Equation Modeling (SEM) through Smart PLS, which allows for the assessment of complex relationships between the variables^35^. SEM is particularly suitable for this study as it accommodates latent constructs, handling multiple interrelated dependencies, and provides robust path coefficient estimation. The path analysis will evaluate the relationships between visionary leadership, digital leadership, knowledge sharing, and digital citizenship in healthcare, ensuring that the model’s reliability and validity are thoroughly tested. This methodology ensures that the study captures the nuanced role of leadership in fostering a digital culture and promoting knowledge sharing in healthcare organizations.

The current study obtained approval from institutional research ethics committee via approval letter (Ref,;No; USTB/Ethics/220 dated 11-07-2024) from the University of Science and Technology Bannu Ethics Committee. As the participation was voluntarily, hence approved informed consent from all the participants were received prior to their participation in the survey.

## Results

### Demographic characteristics of respondents

The demographic profile of the 202 respondents participating in the study provides valuable insights into the workforce’s gender distribution, work experience, educational background, and technological proficiency within the healthcare sector.

Out of the total 202 respondents, 171 are male, which accounts for approximately 84.7% of the sample, while the remaining 31 are female (15.3%). This gender imbalance reflects a male-dominated workforce in the healthcare sector, which may be a factor in how leadership roles and responsibilities, particularly in digital and visionary leadership, are distributed.

In terms of professional experience, 102 respondents (50.5%) have more than 10 years of work experience, indicating a significant portion of seasoned professionals who likely possess in-depth knowledge and insights into leadership and healthcare operations. This is followed by 55 respondents (27.2%) who have between 5 and 10 years of experience, representing mid-career professionals, while 48 respondents (22.3%) have less than 5 years of experience. This spread across different levels of experience is essential for understanding how experience impacts knowledge sharing, leadership adoption, and technological integration in healthcare.

Regarding education, 160 respondents (79.2%) have completed more than 16 years of education, indicating a high level of academic qualifications, typical of healthcare professionals who have undergone advanced training. Additionally, 42 respondents (20.8%) possess higher qualifications, such as postgraduate degrees or professional certifications. This highly educated workforce is likely to be more receptive to advanced digital leadership concepts and technological innovations, which are critical in knowledge sharing and improving healthcare outcomes.

A significant majority of 185 respondents (91.6%) report having access to and expert knowledge of digital technologies, including emerging trends. This suggests that the majority of the workforce is technologically proficient, a crucial factor in the effective implementation of digital leadership and knowledge sharing. The remaining 17 respondents (8.4%) have moderate knowledge of digital technologies, which highlights the need for continuous digital literacy training to ensure full inclusion in the digital transformation of healthcare operations.

In light of the above findings, the majority of respondents have substantial professional experience, high educational qualifications, and strong technological knowledge. This positions them well to contribute to, and benefit from, initiatives aimed at enhancing knowledge sharing through digital and visionary leadership. However, the gender imbalance and the minority with moderate digital proficiency may require targeted interventions to ensure equal participation and opportunities for leadership roles in the healthcare sector.

**Table 1:** Construct validity.

		Item Loading	CA	CR	AVE
Visionary Leadership					
1	My institutional head inspires a clear vision for using digital tools to improve healthcare delivery and patient care outcomes.	0.770	0.703	0.820	0.604
2	My institutional head drives innovation by facilitating the adoption of new digital technologies in healthcare settings.	0.787			
3	My institutional head actively communicates the future vision for technology-driven healthcare, motivating us to align with organizational goals.	0.874			
Digital Leadership					
1	My leader involves me in decision-making processes regarding the use of digital tools and technology in my healthcare role.	0.781	0.830	0.876	0.541
2	My leader encourages me to develop my digital skills, ensuring I stay updated on the latest technological advancements in healthcare.	0.785			
3	My leader provides support and guidance whenever I encounter challenges with digital healthcare systems or tools.	0.785			
4	My leader gives regular feedback on how effectively I am using digital technology in my healthcare role.	0.642			
5	My leader ensures I have access to the necessary digital tools and information to perform my work efficiently.	0.700			
6	My leader promotes the use of innovative digital methods within our department, helping to improve patient care and workflow efficiency.	0.708			
Digital Citizenship					
1	I ensure that all healthcare employees have access to the appropriate digital tools and resources needed to enhance patient care and learning outcomes.	0.826	0.720	0.842	0.641
2	I actively model and promote the ethical, legal, and responsible use of digital tools and technologies within the healthcare environment.	0.798			
3	I encourage the use of digital collaboration tools to facilitate a shared understanding of global healthcare challenges and innovations.	0.776			
4	I foster an organizational culture that supports responsible social interactions related to the use of healthcare technology and digital information.		Item was removed due to poor loading		
Knowledge Sharing					
1	I reward healthcare employees who share their knowledge and best practices, contributing to improved patient care and organizational success.	0.826	0.912	0.938	0.790
2	I encourage knowledge sharing by promoting a culture of openness, learning from mistakes, and focusing on achieving our healthcare objectives.	0.798			
3	I ensure that employees are supported in learning from their experiences, and I tolerate mistakes when they contribute to knowledge growth and innovation in patient care.	0.776			
4	I serve as a mentor, guiding employees in applying their knowledge, and using assessments to help them achieve their professional goals.	0.826			
5	I have implemented structured processes for distributing knowledge across the organization, ensuring that healthcare innovations and best practices are shared effectively		Item was removed due to poor loading		

The construct validity results presented in Table 1 provide insights into the reliability and validity of the variables measured in the study. These variables include Visionary Leadership, Digital Leadership, Digital Citizenship, and Knowledge Sharing. The results are evaluated based on item loadings, Cronbach’s alpha (CA), Composite Reliability (CR), and Average Variance Extracted (AVE), which indicate the consistency and validity of the constructs^36^.

The Visionary Leadership construct consists of three items, with item loadings ranging from 0.770 to 0.874, indicating strong individual contributions to the construct. The Cronbach’s alpha (CA) of 0.703 suggests acceptable internal consistency, while the Composite Reliability (CR) is 0.820, demonstrating a high level of construct reliability. The AVE value of 0.604 exceeds the minimum threshold of 0.5, indicating good convergent validity^37^. This suggests that leaders in healthcare institutions effectively inspire a clear vision for using digital tools, communicate future visions, and facilitate innovation by adopting new technologies.

The Digital Leadership construct comprises six items, with item loadings ranging from 0.642 to 0.785. The Cronbach’s alpha of 0.830 indicates high internal consistency^38^, and the Composite Reliability (CR) of 0.876 confirms strong reliability. The AVE of 0.541 is also above the acceptable threshold, confirming adequate convergent validity. These results reflect that healthcare leaders actively involve employees in decision-making processes, encourage digital skills development, and provide necessary support and resources for efficient digital tool usage.

Digital Citizenship is assessed using three items after one item was removed due to poor loading. The item loadings range from 0.776 to 0.826, reflecting robust contributions from the remaining items. The Cronbach’s alpha of 0.720 and Composite Reliability of 0.842 indicate good reliability^38^. The AVE of 0.641 shows strong convergent validity, suggesting that healthcare leaders promote ethical and responsible digital tool use and facilitate global collaboration through digital tools.

The Knowledge Sharing construct has five items, with two items removed due to poor loading. The remaining items exhibit loadings between 0.776 and 0.826. Cronbach’s alpha is high at 0.912, and the Composite Reliability is 0.938, showing excellent reliability. The AVE of 0.790 further confirms strong convergent validity^37,38^. This indicates that healthcare leaders reward employees for knowledge sharing, promote a culture of openness, and mentor employees to apply their knowledge effectively (See Table 1).

Overall, the constructs show good reliability and validity, confirming that the items effectively measure Visionary Leadership, Digital Leadership, Digital Citizenship, and Knowledge Sharing in healthcare settings. These results support the idea that leadership plays a pivotal role in promoting digital innovation, responsible digital behavior, and knowledge sharing within healthcare organizations.

**Table 2:** Discriminant validity.

	DCZ	DLR	KNS	VSL
DCZ	0.800			
DLR	0.640	0.736		
KNS	0.628	0.736	0.889	
VSL	0.539	0.552	0.609	0.777
HTMT				
DCZ				
DLR	0.809			
KNS	0.768	0.834		
VSL	0.769	0.736	0.768	

The Fornell-Larcker Criterion is a widely used method for evaluating discriminant validity in structural equation modeling. Discriminant validity assesses whether constructs that should not be related are, in fact, distinct from each other. According to this criterion, the square root of the Average Variance Extracted (AVE) for each construct should be greater than the correlation of that construct with any other construct in the model.

For example, the square root of the AVE for DCZ (Digital Citizenship) is 0.800, which is greater than its correlation with DLR (Digital Leadership) at 0.640, KNS at 0.628, and VSL at 0.539. This indicates strong discriminant validity for the DCZ construct^38^. Similarly, the square root of the AVE for the other constructs (DLR = 0.736, KNS = 0.889, VSL = 0.777) is also greater than their respective correlations with other constructs, confirming that each construct is distinct from the others (see Table 2).

The HTMT criterion is a more modern approach for assessing discriminant validity, considered stricter than the Fornell-Larcker Criterion. HTMT calculates the ratio of the average correlations of items across constructs to the average correlations of items within the same construct. An HTMT value below 0.90 generally indicates good discriminant validity^39^.

The values for the constructs are all below 0.90, with the highest being 0.834 between DLR and KNS, suggesting that the constructs have good discriminant validity.

For example, the HTMT between DCZ and DLR is 0.809, between KNS and VSL is 0.768, and between DLR and VSL is 0.736, all of which are acceptable according to the HTMT threshold^39^.

Both the Fornell-Larcker Criterion^39^ and the HTMT^40^ results confirm that the constructs in the study (Digital Citizenship, Digital Leadership, Knowledge Sharing, and Visionary Leadership) exhibit strong discriminant validity. This means that the constructs are distinct and do not overlap significantly in their measurement, ensuring the accuracy and reliability of the study’s measurement model.

**Table 3:** Hypothesis testing results.

Hypothesis	Relationship	Beta	T	*P* values	Decision
H1	DCZ -> KNS	0.190	3.199	0.001	Supported
H2	DLR -> DCZ	0.492	8.577	0.000	Supported
H3	DLR -> KNS	0.482	7.475	0.000	Supported
H4	VSL -> DCZ	0.268	4.861	0.000	Supported
H5	VSL -> KNS	0.240	4.543	0.000	Supported
H6	DLR -> DCZ -> KNS	0.093	2.867	0.004	Supported
H7	VSL -> DCZ -> KNS	0.051	2.484	0.013	Supported

**Fig. 2:**
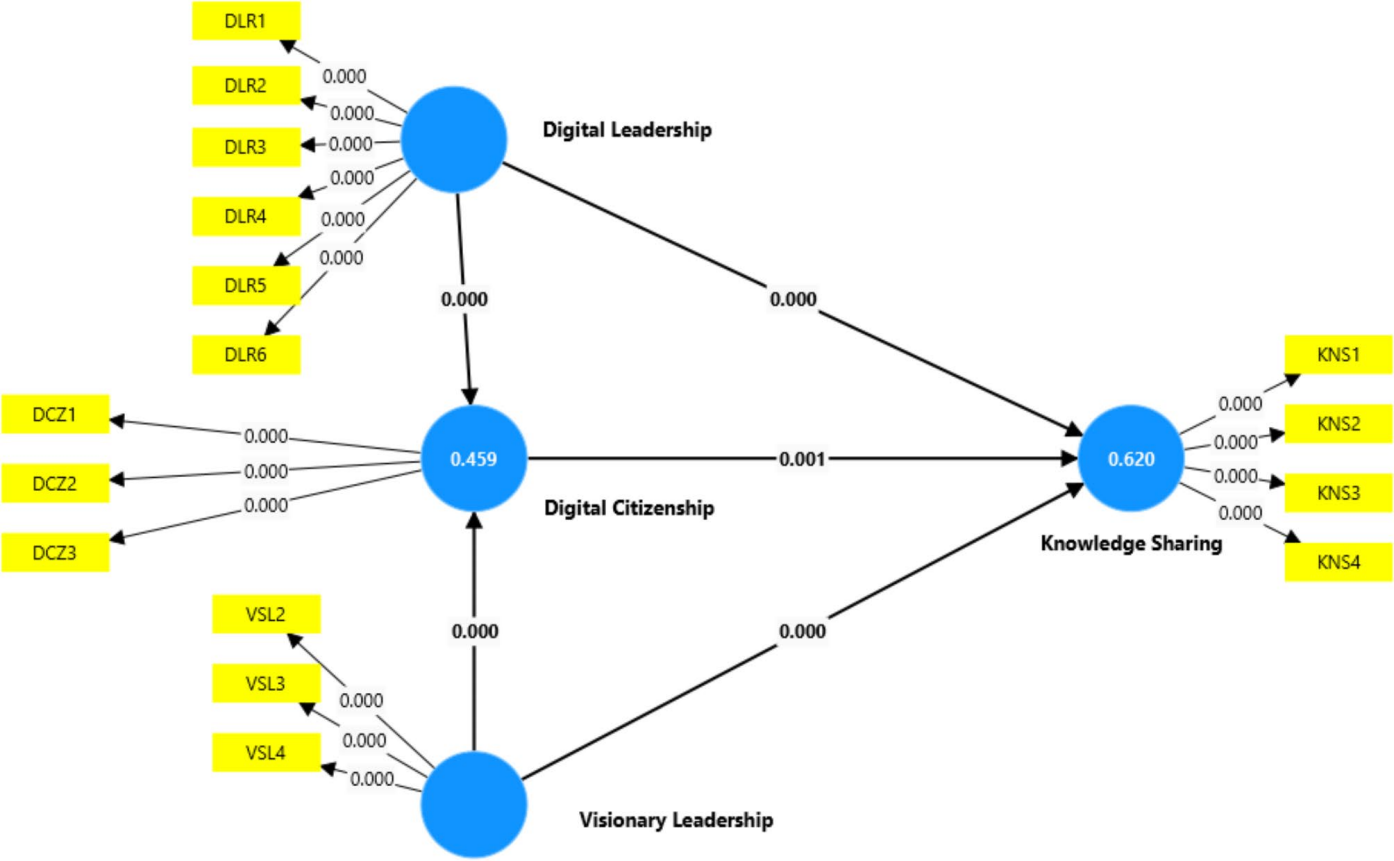
Path Coefficient through Smart PLS.

The relationship between Digital Citizenship (DCZ) and Knowledge Sharing (KNS) is positive, with a Beta value of 0.190, indicating a moderate influence. The T-value of 3.199, well above the threshold of 1.96, confirms statistical significance. The P-value of 0.001, being less than 0.05 (see Fig. 2), further supports the hypothesis (Hypothesis 1: DCZ -> KNS) that a strong digital citizenship culture in healthcare encourages employees to share knowledge, enhancing overall organizational learning (see Table 3).

Furthermore, Digital Leadership (DLR) has a substantial impact on Digital Citizenship (DCZ), as indicated by the Beta of 0.492, showing a strong relationship. The T-value of 8.577 far exceeds the significance threshold, and the P-value of 0.000 confirms that this relationship is highly significant. This result demonstrates that leaders who engage in digital leadership practices strongly foster a culture of responsible digital use within healthcare organizations, hence accept (H2: DLR -> DCZ).

The Beta value of 0.482 suggests a robust positive effect of Digital Leadership (DLR) on Knowledge Sharing. A T-value of 7.475 indicates that this effect is statistically significant, and the P-value of 0.000 reinforces the hypothesis that (H3; DLR -> KNS). Leaders who involve employees in digital decision-making and provide technological support enhance employees’ willingness and ability to share knowledge in healthcare settings.

Visionary Leadership has a positive and significant influence on Digital Citizenship (see Fig. 2), as seen by the Beta of 0.268, which shows a moderate relationship. The T-value of 4.861 signifies strong statistical support, and the P-value of 0.000 confirms the hypothesis (H4: VSL -> DCZ). Visionary leaders in healthcare who inspire and guide digital innovation significantly contribute to fostering a culture of responsible digital behavior.

Visionary Leadership also has a moderate and significant effect on Knowledge Sharing, as indicated by the Beta of 0.240. The T-value of 4.543, coupled with a P-value of 0.000, shows that visionary leadership in healthcare positively influences employees’ willingness to share knowledge, driven by a clear and inspiring digital vision and leads to accept (H5: VSL -> KNS).

This mediation hypothesis reveals that Digital Citizenship partially mediates the relationship between Digital Leadership and Knowledge Sharing (KNS). The Beta of 0.093 shows a small but significant mediation effect. The T-value of 2.867 and P-value of 0.004 confirm the mediation, highlighting the importance of digital citizenship in enhancing knowledge sharing through effective digital leadership. Hence (H6: DLR -> DCZ -> KNS) is also accepted.

The mediation role of Digital Citizenship in the relationship between Visionary Leadership (VSL) and Knowledge Sharing (KNS) is confirmed by a Beta of 0.051. Although the effect is smaller, the T-value of 2.484 and P-value of 0.013 indicate that Digital Citizenship partially mediates this relationship^41^. Visionary leaders’ focus on fostering digital citizenship can thus further enhance the sharing of knowledge within healthcare organizations that leads to accept (H7: VSL -> DCZ -> KNS).

## Discussion

The results indicate that digital citizenship plays a constructive role in knowledge sharing in hospitals. This implies that there is a positive relationship between responsible employee behaviors in the virtual environment and knowledge sharing. This concurs with research showing that digitally responsible culture improves communication and knowledge sharing, which are critical for increasing knowledge exchange^5^. In the healthcare field where the application of the information technology is advancing, supporting the principles of digital citizenship contributes to the effective dissemination of knowledge, and thereby contributes to enhancing patient care as well as enhance HCE capabilities.

Furthermore, it is evident that digital leadership significantly correlates to digital Citizenship, which implies that leaders hold an essential responsibility of leading people into proper discrete behavior that forms part of health care organizations. This result complements earlier studies that define leadership as an important factor in establishing a digitally responsible workforce^42^. Supervisors or managers who promote and model digital innovation and appropriate use of technology facilitates the organization’s responsible digital citizenship^5^. When it comes to healthcare, a sector that relies heavily on technology in healthcare and management^26^, having digital vision and leadership means that an acceptable culture towards use of ethical technology in meeting the intended organizational goals for the enhancement of patient care delivery is enhanced.

The above findings indicate that digital leadership has a positive influence on the levels of knowledge sharing. This corroborates findings from Anwar and Saraih^5^ who found out that leaders who are pro-active in the adoption of digital agenda create an environment that supports sharing of knowledge. In terms of knowledge sharing where leaders of care organizations have the power to put patients’ lives at risk by not sharing this knowledge effectively, such leaders who espouse some form of digital innovation allow their teams to share the knowledge effectively. This paper therefore underscores the role of digital leadership in the kind of collaborative practices in the health care systems.

This study shows that visionary leadership is important in enhancing and encouraging the development of digital citizenship. Managers who paint a positive picture about the use of technology in future encourage constructive use of technology among the workers^43^. In this regard, the debate of Tellis^44^ supports that visionary leaders establish a collective values system in regards to the strategic role of IT resulting in increased ethical use of technologies. In healthcare, charismatic leadership that supports the application of innovative technology can inspire employees to embrace digital citizenship behaviors, which, in turn, enhances organizational productivity and improves the efficiency of health delivery.

The fact that visionary leadership has a direct positive impact with knowledge sharing^45^ shows that leadership with a positive vision can enhance a culture of knowledge sharing. This is in consonance with the previous studies^45,46^, which corroborates that visionary leadership leads to the harnessing of employee contribution to organizational knowledge through the sharing of goals. It is notably true that in healthcare when great innovation and a good understanding of current theories and practices remain critical for delivering quality and holistic patient care, visionary leaders establish an environment where the employees conceptualize and contribute toward change.

This hypothesis focuses on mediating effect of digital citizenship in the relationship between digital leadership and knowledge sharing. This suggests that knowledge sharing in the workplace is a function of effective responsible digital behaviors that digital leadership fosters^47^. Similarly Anwar and Saraih^5^, also arguing that digital citizenship is an appropriate mechanism to be use as digital strategy to enhances co- working and sharing of information. In the context of healthcare, it illustrates how digital leaders who prioritize ethical technology adoption and application create an environment conducive to knowledge dissemination, ultimately enhancing both patient care and organizational learning.

The results also indicate that digital citizenship mediate the relationship between visionary leadership and knowledge sharing. Leaders who establish a positive paradigm for technology utilization^26^ foster a culture of knowledge sharing among employees^1^. This aligns with the findings of Anwar and Saraih^5^ which indicate that when visionary leadership is implemented, it promotes responsible digital behavior and subsequently enhances knowledge sharing within the organization. This dynamic is useful in the healthcare industry to guarantee that not only does the staff embraces change and incorporate new technologies into their work environment, but also utilize these technologies to exchange potential cardinality of use value related comprehensive information that may enhance patient care and business efficiency.

## Conclusion

In the context of Pakistan, this study reveals the crucial role of both visionary and digital leadership in transforming the healthcare sector. As digital technologies continue to reshape healthcare practices, effective leadership becomes indispensable in guiding organizations through this transition. Visionary leaders, by articulating a clear and future-oriented technological vision, help create a culture where employees are motivated to embrace change and innovation. In contrast, the digital leaders have a crucial responsibility for helping the healthcare workers to acquire the relevant digital competencies needed to address the rising requirements of patients’ care and their career advancement. Since this model of leadership encourages knowledge sharing, it is effective in promoting learning, better decision-making and ultimately patients’ welfare.

The leadership is crucial in Pakistan because as a developing country it has to overcome a number of challenges that are specific to the healthcare sector i.e. the shortages of resources, bureaucracy, and lack of adequate technology. The study shows that it is possible to substantially enhance the effectiveness of an organization, as well as the quality of the care provided to the patients, through the establishment of leaders’ ethical and responsible approach to the integration of the digital technologies with the aim of developing collaborative knowledge-sharing environments. By supporting healthcare workers and investing in the betterment of the healthcare personnel these leaders are also helping in creating a stronger and more flexible healthcare system.

This study raises concern on how leadership development programs could incorporate skills in both digital and visionary leadership to meet the current needs. Such initiatives would be able to extend knowledge and practical approaches to deal with the challenges that are bound to come with technological advancement in the delivery of healthcare services, as well as make sure that information technology is embedded responsibly and in a right manner. From a theoretical perspective, the results shown that the digital citizenship behavior provided an important link between the digital/visionary leadership and the extent to which employees engaged in knowledge sharing. The digital leadership promotes the use of advanced tools for enhancing the rates of productivity among employees and promoting the flow of information, which is vital to the building of knowledge sharing culture^27^. Visionary leadership adds to this by educating the employees to embrace a certain vision, match their specific self-interests to the organization strategic objectives and consequently inspiring the employees to behave proactively about knowledge sharing^28,29^. Pro-organizational citizenship behavior that is a discretionary activity that goes beyond expected organizational roles is more prevalent in organizations in which leadership promotes organizational citizenship behavior and where organizational membership leads to perceived oneness with other members. It remains especially the case when the visionary leadership is in place, which fosters the shared sense of organizational mission; or the digital leadership that provides the technological backing to various collaborative initiatives and activities. Altogether, such leadership practices contribute to the climate in which knowledge is spread without imposing, as well as improve innovative and learning processes by collaboration and proactive actions are encouraged^30^. Such synergistic effect underlines the mediating role of the digital citizenship behavior in facilitating the constructive and purposeful sharing of knowledge by the employees. Thus, in the long run, this approach might eventually bring the integration of technology, traditional healthcare practices and better adaptation to the needs of a technological environment to Pakistani healthcare system, making it adaptable and competitive with the global counterparts.

The study on visionary and digital leadership in Pakistan’s healthcare sector has several limitations. The study fails to adapt to distinct healthcare difficulties between Pakistan’s urban centers and rural regions. This emphasis on leadership styles fails to consider crucial aspects such as organizational culture together with employee morale and external socio-economic factors which determine healthcare delivery effectiveness. This study clearly demonstrates how leadership stands essential for digital integration yet skips examining distinct hurdles healthcare organizations encounter when implementing new technologies such as physical infrastructure limitations and employee resistance to change. Practical implementation of research findings faces limitations due to the use of theoretical frameworks because real-world situations rarely present themselves as neat and precise as theory does. The current study needs additional empirical testing to establish clear connections between digital citizenship conduct and knowledge exchange behavior. The proposed leadership development programs present value but may experience obstacles in implementation because they depend on sufficient resources as well as political backing. No comprehensive analytical approach exists to understand how these leadership approaches evolve patient care and organizational effectiveness over time within Pakistan’s developing healthcare system.25.

According to the current study, the following recommendations are suggested for future researchers as outlined below;

It is advised that future research should compare the impacts of the two types of leadership in sectors other than healthcare for instance in education and banking sectors in an attempt to identify the differences that exist in leadership in different sectors of Pakistan.

Future studies could use longitudinal data to analyses the changes in the employment of digital technologies and visionary leadership characteristics to understand the long-term impact on knowledge sharing and caring organization’s performance.

Subsequent research should explore the concerns that relate to the enactment of digital technology support for leadership and knowledge management. It might be useful to try to understand how and in what ways restricted availability of technology in leadership in Pakistan and especially in rural areas.

Research with the focus on how the cultural aspects such as power distance and the hierarchical leadership style that is traditional in Pakistan affects the aspects of digital leadership as well as the digital citizenship in the healthcare organizations would assist in taking the research even further.

Research should also look into how the training of professional development programs through healthcare leaders in Pakistan can improve the skills of digital leadership especially in the low resource areas in the country where access to most of the new innovations is very rare.

More emphasis can be placed in future research on the perceptions of the employees on leadership concerning the incumbent’s role in enhancing the workers’ knowledge of digitization in the health sector and information-sharing.

Lastly, the role of government policies in supporting digital transformation in Pakistan’s healthcare sector should be explored, with an emphasis on how leadership and knowledge sharing align with national digital health strategies.

## Data Availability

The data that support the findings of this study are available from the corresponding author upon reasonable request.

## References

[1] Sher, P. J. & Lee, V. C. Information technology as a facilitator for enhancing dynamic capabilities through knowledge management. *Inf. Manag*. **41** (8), 933–945 (2004).

[2] Caridi-Zahavi, O., Carmeli, A. & Arazy, O. The influence of ceos’ visionary innovation leadership on the performance of high‐technology ventures: the mediating roles of connectivity and knowledge integration. *J. Prod. Innov. Manag*. **33** (3), 356–376 (2016).

[3] Paroutis, S. & Al Saleh, A. Determinants of knowledge sharing using web 2.0 technologies. *J. Knowl. Manag*. **13** (4), 52–63 (2009).

[4] Bresciani, S., Ferraris, A., Romano, M. & Santoro, G. Digital leadership. In: Digital Transformation Management for Agile Organizations: A Compass To Sail the Digital World. Emerald Publishing Limited; 97–115. (2021).

[5] Anwar, S. & Saraih, U. N. Digital leadership in the digital era of education: enhancing knowledge sharing and emotional intelligence. Int. J. Educ. Manag. 10.1108/IJEM-11-2023-0540 (2024).

[6] Madugu, U. & Manaf, H. A. Visionary leadership and individual academic staff performance: the mediating influence of knowledge sharing. *Manag Res. Spectr.* **9** (2), 60–66 (2019).

[7] Olan, F. et al. Artificial intelligence and knowledge sharing: contributing factors to organizational performance. *J. Bus. Res.* **145**, 605–615 (2022).

[8] Wei, H. & Horton-Deutsch, S. *Visionary Leadership in Healthcare.* (Sigma Theta Tau, 2022).

[9] VanDoorn, G. & Eklund, A. Face to Facebook: social media and the learning and teaching potential of symmetrical, sychronous communication. *J. Univ. Teach. Learn. Pract.* **10**(1). (2013).

[10] Cao, X., Guo, X., Vogel, D. & Zhang, X. Exploring the influence of social media on employee work performance. *Internet Res.* **26** (2), 529–545 (2016).

[11] Wang, G. et al. Development of metaverse for intelligent healthcare. *Nat. Mach. Intell.* **4** (11), 922–929 (2022). 36935774 10.1038/s42256-022-00549-6 PMC10015955

[12] Mozumder, M. A. I. et al. Metaverse for digital anti-aging healthcare: an overview of potential use cases based on artificial intelligence, blockchain, IoT technologies, its challenges, and future directions. *Appl. Sci.* **13** (8), 5127 (2023).

[13] Abdulmuhsin, A. A., Owain, H. O. & Alkhwaldi, A. F. Understanding the academic use of KM-driven metaverse technology: insights from medical colleges. *J. Sci. Technol. Policy Manag* (2024).

[14] Anwar, S. & Saraih, U. N. Digital leadership in the digital era of education: enhancing knowledge sharing and emotional intelligence. *Int. J. Educ. Manag.* (2024).

[15] Ribble, M. & Park, M. *The Digital Citizenship Handbook for School Leaders: Fostering Positive Interactions Online.* (International Society for Technology in Education, 2022).

[16] Baydar, F. The role of educational leaders in the development of students’ technology use and digital citizenship. *Malaysian Online J. Educ. Technol.* **10** (1), 32–46 (2022).

[17] Formosa, P., Wilson, M. & Richards, D. A principlist framework for cybersecurity ethics. *Comput. Secur.* **109**, 102382 (2021).

[18] Luttrell, R. *Social Media: How To Engage, Share, and Connect.* (Rowman & Littlefield, 2021).

[19] Rasel, M. D., Shovon, R. B. & Islam, M. A. Ethical Data-Driven innovation: integrating cybersecurity analytics and business intelligence for responsible governance. *J. Environ. Sci. Technol.* **2** (2), 143–168 (2023).

[20] Khan, H. U., Ali, Y., Khan, F. & Al-Antari, M. A. A comprehensive study on unraveling the advances of immersive technologies (VR/AR/MR/XR) in the healthcare sector during the COVID-19: challenges and solutions. *Heliyon* **10**(15). (2024). 10.1016/j.heliyon.2024.e35037 PMC11328097 39157361

[21] Lowe, S. Celebrating success—visionary leadership recognising fit for health in the delivery of rural and remote primary health care services. (2009).

[22] Omboni, S., Caserini, M., Coronetti, C. & Telemedicine M-health in hypertension management: technologies, applications and clinical evidence. *High. Blood Press. Cardiovasc. Prev.* **23**, 187–196 (2016). 27072129 10.1007/s40292-016-0143-6

[23] Phong, L. B. & Son, T. T. The link between transformational leadership and knowledge sharing: mediating role of distributive, procedural and interactional justice. *J. Inf. Knowl. Manag*. **19** (03), 2050020 (2020).

[24] Anselmann, V. & Mulder, R. H. Transformational leadership, knowledge sharing and reflection, and work teams’ performance: A structural equation modelling analysis. *J. Nurs. Manag*. **28** (7), 1627–1634 (2020). 32754940 10.1111/jonm.13118

[25] Ahmed, M. J. et al. CardioGuard: AI-driven ECG authentication hybrid neural network for predictive health monitoring in telehealth systems. *SLAS Technol.* **29** (5), 100193 (2024). 39307457 10.1016/j.slast.2024.100193

[26] Alanazi, A. T. Digital leadership: attributes of modern healthcare leaders. *Cureus* **14**(2). (2022). 10.7759/cureus.21969 PMC8906562 35282530

[27] Sousa, M. J. & Rocha, Á. Digital learning: developing skills for digital transformation of organizations. *Futur Gener Comput. Syst.* **91**, 327–334 (2019).

[28] Bass, B. M. & Avolio, B. J. Transformational leadership and organizational culture. *Public. Adm. Q.* 112–121. (1993).

[29] Bass, B. M. & Bass Bernard, M. Leadership and performance beyond expectations. (1985).

[30] Podsakoff, P. M., MacKenzie, S. B., Paine, J. B. & Bachrach, D. G. Organizational citizenship behaviors: A critical review of the theoretical and empirical literature and suggestions for future research. *J. Manage.* **26** (3), 513–563 (2000).

[31] Donate, M. J. & de Pablo, J. D. S. The role of knowledge-oriented leadership in knowledge management practices and innovation. *J. Bus. Res.* **68** (2), 360–370 (2015).

[32] AlAjmi, M. K. The impact of digital leadership on teachers’ technology integration during the COVID-19 pandemic in Kuwait. *Int. J. Educ. Res.* **112**, 101928 (2022). 35153373 10.1016/j.ijer.2022.101928 PMC8825317

[33] Thannimalai, R. & Raman, A. Principals technology leadership and teachers technology integration in the 21st century classroom. *Int. J. Civ. Eng. Technol.* **9** (2), 177–187 (2018).

[34] Claassen, K., Dos Anjos, D. R., Kettschau, J. & Broding, H. C. How to evaluate digital leadership: a cross-sectional study. *J. Occup. Med. Toxicol.* **16**, 1–8 (2021). 34598724 10.1186/s12995-021-00335-x PMC8485107

[35] Hair, J. F. Jr, Matthews, L. M., Matthews, R. L. & Sarstedt, M. PLS-SEM or CB-SEM: updated guidelines on which method to use. *Int. J. Multivar. Data Anal.* **1** (2), 107–123 (2017).

[36] Memon, M. A. et al. PLS-SEM statistical programs: a review. *J. Appl. Struct. Equ Model.* **5** (1), 1–14 (2021).

[37] Cheah, J. H., Sarstedt, M., Ringle, C. M., Ramayah, T. & Ting, H. Convergent validity assessment of formatively measured constructs in PLS-SEM: on using single-item versus multi-item measures in redundancy analyses. *Int. J. Contemp. Hosp. Manag* (2018).

[38] Cheung, G. W., Cooper-Thomas, H. D., Lau, R. S. & Wang, L. C. Reporting reliability, convergent and discriminant validity with structural equation modeling: A review and best-practice recommendations. *Asia Pac. J. Manag* 1–39. (2023).

[39] Ab Hamid, M. R., Sami, W. & Sidek, M. H. M. Discriminant validity assessment: Use of Fornell & Larcker criterion versus HTMT criterion. In: Journal of Physics: Conference Series. 12163. ((IOP Publishing, 2017).

[40] Roemer, E., Schuberth, F. & Henseler, J. HTMT2–an improved criterion for assessing discriminant validity in structural equation modeling. *Ind. Manag Data Syst.* (2021).

[41] Kayani, M. B., Shafique, K. & Ali, M. How does leadership bring individual creativity? A mediation and moderation analysis. *Int. J. Work Innov.* **3** (4), 382–402 (2023).

[42] Tigre, F. B., Curado, C. & Henriques, P. L. Digital leadership: A bibliometric analysis. *J. Leadersh. Organ. Stud.* **30** (1), 40–70 (2023).

[43] Laukka, E., Pölkki, T. & Kanste, O. Leadership in the context of digital health services: A concept analysis. *J. Nurs. Manag*. **30** (7), 2763–2780 (2022). 35942802 10.1111/jonm.13763 PMC10087820

[44] Tellis, G. J. Disruptive technology or visionary leadership? *J. Prod. Innov. Manag*. **23** (1), 34–38 (2006).

[45] Rajagopal, S. *Impact of Visionary Leadership, Partner Alignment, and Knowledge Sharing on Partner Performance.* (Drexel University, 2021).

[46] Zhou, L., Zhao, S., Tian, F., Zhang, X. & Chen, S. Visionary leadership and employee creativity in China. *Int. J. Manpow.* **39** (1), 93–105 (2018).

[47] Gu, A., Nawaz, A., Abbas, S. & Lv, B. Enhancing organizational performance through knowledge-oriented leadership: the neglected role of employee creative work behavior and digital citizenship behavior in IT industry. Kybernetes. (2024).

